# The complete chloroplast genome of *Camellia flava* (Pitard) Sealy, a golden camellia of Vietnam

**DOI:** 10.1080/23802359.2024.2392741

**Published:** 2024-08-21

**Authors:** Yunxia Tang, Xingwen Zhou, Mengyao Zhu, BingBing He, Changjie Jiang, Guochang Ding

**Affiliations:** a College of Landscape Architecture and Art, Fujian Agriculture and Forestry University, Fuzhou, China; b College of Architecture and Planning, Fujian University of Technology; c Golden Camellia Park of Nanning, Guangxi Zhuang Autonomous Region, Nanning, China

**Keywords:** Theaceae, ornamental plant, phylogenetic analysis

## Abstract

*Camellia flava* (Pit.) Sealy 1949 is a rare and precious species with golden flowers, which hold important ornamental and breeding values. In this study, the complete chloroplast genome of *C. flava* is reported for the first time. The chloroplast genome exhibits a typical quadripartite structure with a total length of 156,670 bp and a GC content of 37.32%, including a large single-copy region (86,250 bp), a small single-copy region (18,292 bp), and a pair of inverted repeat regions (26,064 bp). A total of 133 genes, including 88 protein-coding genes, 37 tRNA genes, and 8 rRNA genes were annotated. The phylogenetic analysis revealed a close relationship between *C. flava* and *C. tamdaoensis*. The chloroplast genome sequence of *C. flava* serves as a valuable resource for further breeding research and genetic phylogenetic studies.

## Introduction

**Figure 1.**
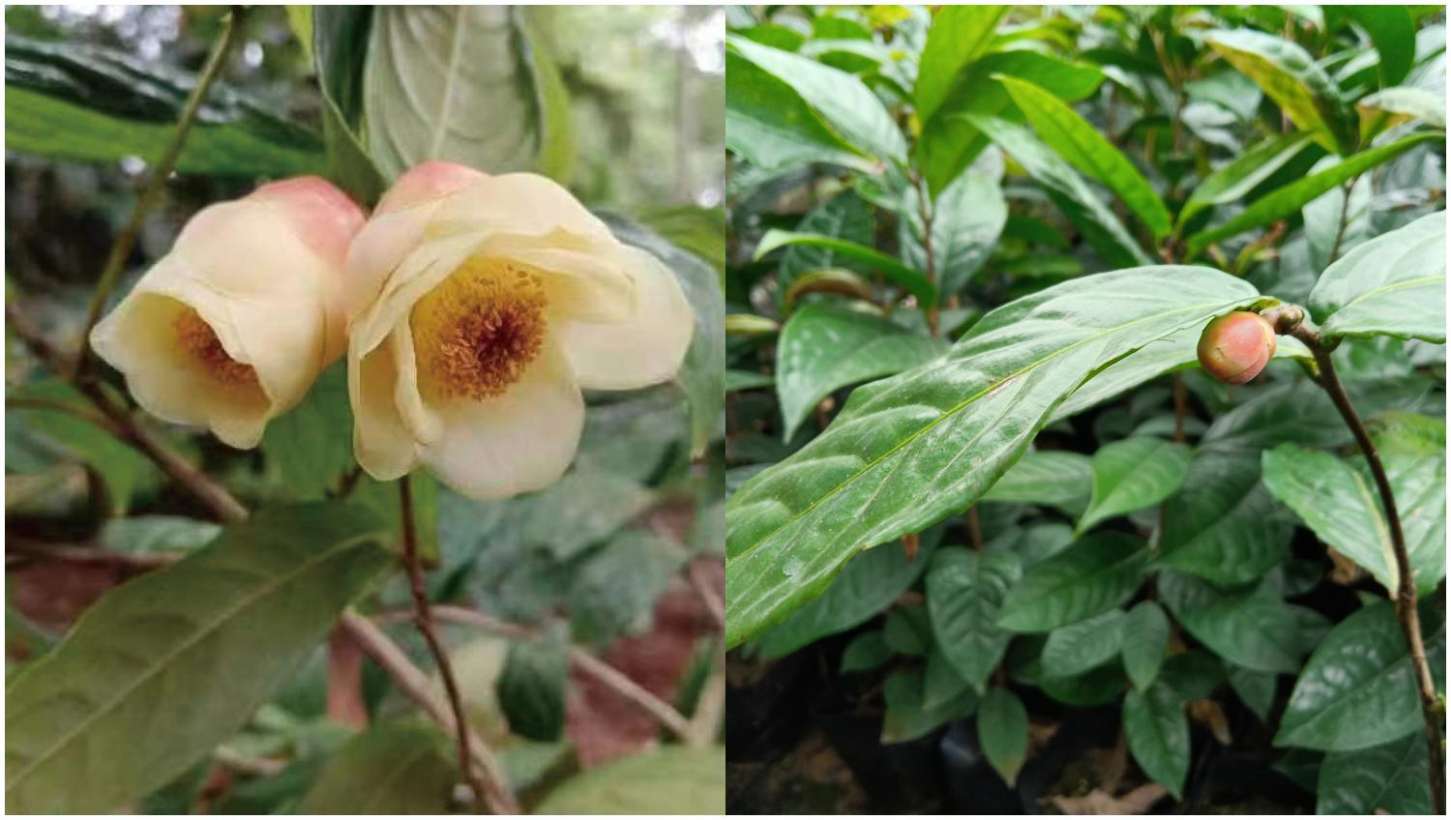
Photograph of *Camellia flava* (the photograph was taken by prof. Guochang Ding on April 1, 2023). *C. flava* leaves ovate-oblong, base shallow heart-shaped, leaf margin sparse serrated; the flower is golden yellow in color, with single or 2–3 clustered branches or leaf axils, bell-shaped, 10–13 petals.

*Camellia flava* (Pit.) Sealy 1949, belonging to the genus *Camellia* L., is one species of golden camellias. It was first described by C.J. Pitard as *Thea flava* Pit. in 1910, then renamed by J.R. Sealy when the genus *Thea* was included in *Camellia* (Sealy 1958). This species is merely distributed in Vietnam, and usually grows in mixed forests of limestone mountains at altitudes of 250–600 meters. It holds significant horticultural and ornamental value because of its bright golden yellow flowers (Figure 1). Golden camellias often possess tea polyphenols and tea polysaccharides, which have high edible and medicinal values (He et al. 2018). However, due to the narrow distribution and small population of *C. flava*, there has been little research on it and the chloroplast genome has remained unknown. In this study, the complete chloroplast genome of *C. flava* was assembled and annotated based on Illumina double-end sequencing data, which would be beneficial for further breeding and utilization of this species.

## Materials and methods

Young leaves were collected from cultivated species from the nursery of Yulin Normal University. (N 22.67, E 110.19), and the voucher specimens were preserved in the Laboratory of Plant Specimen of Fujian University of Science and Technology (voucher number: ST202304011, Contact: Guochang Ding, e-mail: yunxtang0319@163.com). We used an improved CTAB (Doyle and Doyle 1987) method to extract the total DNA of the sample. After that, the extracted total DNA was sent to BGI Technology Service Co., Ltd. (Wuhan, China) to construct a library, and the genome was sequenced using the Illumina HiSeq 4000 sequencing platform with a paired-end read length of 150 bp. The number of reads generated from sequencing was 32,021,479, and 438,786 reads were used to assemble the plastid genome. The chloroplast genome sequence was assembled using GetOrganelle v.3.11.0 (Jin et al. 2020). The parameters applied to the plastome assembly were -w 95 -R 20 -k 21, 35, 45, 55, 65, 75 -F embplant_pt. The assembled chloroplast genome was annotated using PGA (Qu et al. 2019), and the start codon and stop codon were manually adjusted using Geneious Prime (Kearse et al. 2012). CPGview (http://www.1kmpg.cn/cpgview) (Liu et al. 2023) was employed to generate genome maps of cis-spliced and trans-spliced genes. The assembled chloroplast genome and its detailed annotations were submitted to GeneBank with the accession number OR605723.

To explore the phylogenetic position of *C. flava*, 35 chloroplast genome sequences were downloaded from GenBank, which contained 29 *Theaceae* species and six other species (*Apterosperma oblata* KY406751, *Gordonia fruticosa* NC035700, *Polyspora speciosa* ON755227, *Po. tonkinensis* ON755228, *Pyrenaria diospyricarpa* MF179488, *Py. menglaensis* KY406747) as outgroups, the six outgroups species are all from *Theaceae* (Supplement Table S1). The whole chloroplast genome sequences were aligned using MAFFT v7.505 (Katoh and Standley 2016). Phylogenetic analysis was performed with maximum likelihood (ML) methods. ML analysis was implemented in RAxML-HPC v. 8.2.10 (Stamatakis 2014) on CIPRES Science Gateway v. 3.3 (Miller et al. 2010), with the TVM + F + I being the best-fit model by ModelFinder (Kalyaanamoorthy et al. 2017) and 1000 bootstrap replicates.

## Results

**Figure 2.**
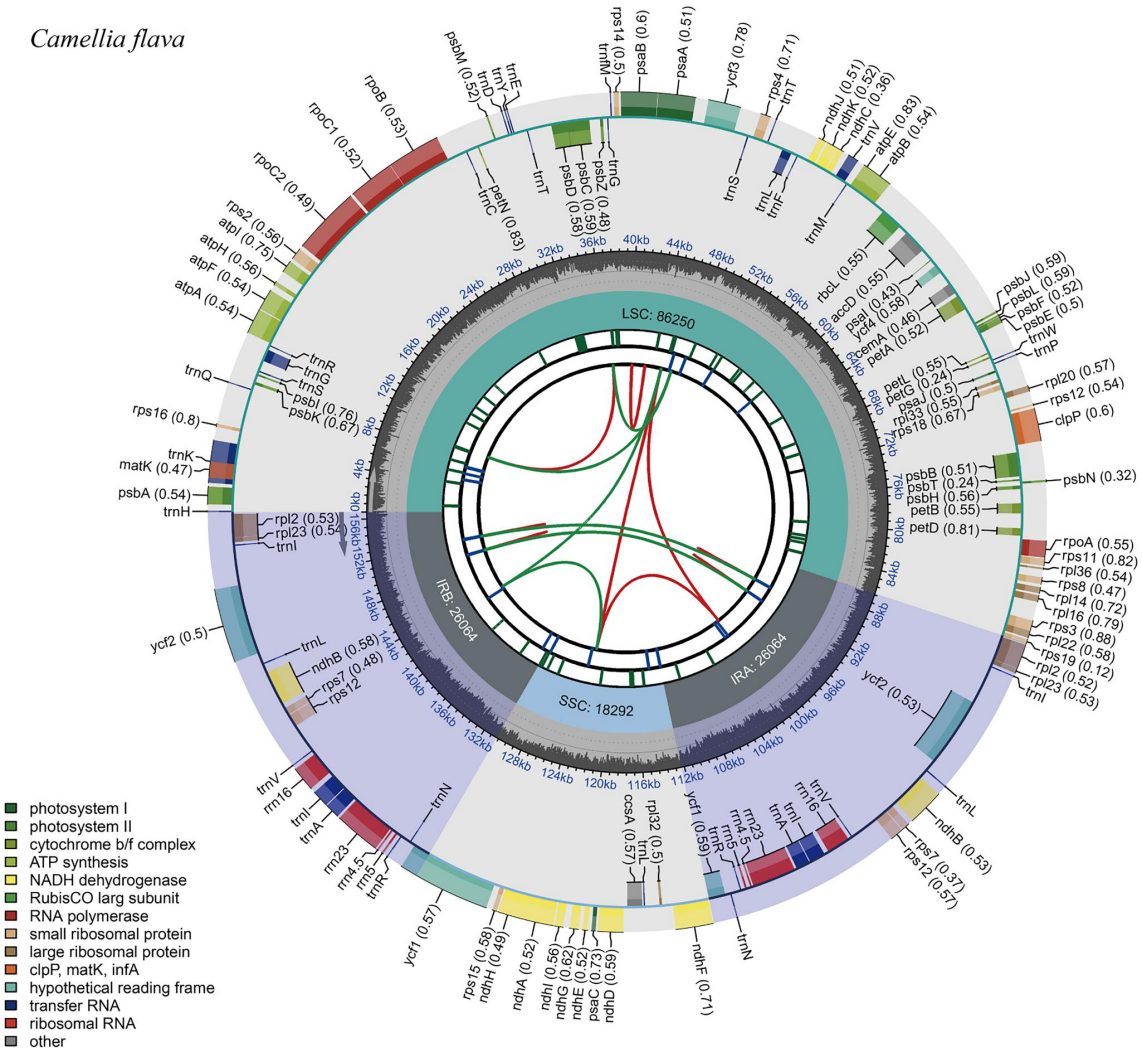
The chloroplast genome map of *Camellia flava*. The species name is shown in the top left corner. The map contains six tracks. From the center outward, the first track shows the dispersed repeats. The dispersed repeats consist of direct (D) and Palindromic (P) repeats, connected with red and green arcs. The second track shows the long tandem repeats as short blue bars. The third track shows the short tandem repeats or microsatellite sequences as short bars with different colors. The colors, the type of repeat they represent, and the description of the repeat types are as follows. Black: c (complex repeat); green: p1 (repeat unit size = 1); yellow: p2 (repeat unit size = 2); purple: p3 (repeat unit size = 3); blue: p4 (repeat unit size = 4); orange: p5 (repeat unit size = 5); red: p6 (repeat unit size = 6). The small single-copy (SSC), inverted repeat (IRa and IRb), and large single-copy (LSC) regions are shown on the fourth track. The GC content along the genome is plotted on the fifth track. The genes are shown on the sixth track. Genes are color-coded by their functional classification. The transcription directions for the inner and outer genes are clockwise and anticlockwise, respectively. The functional classification of the genes is shown in the bottom left corner.

**Figure 3.**
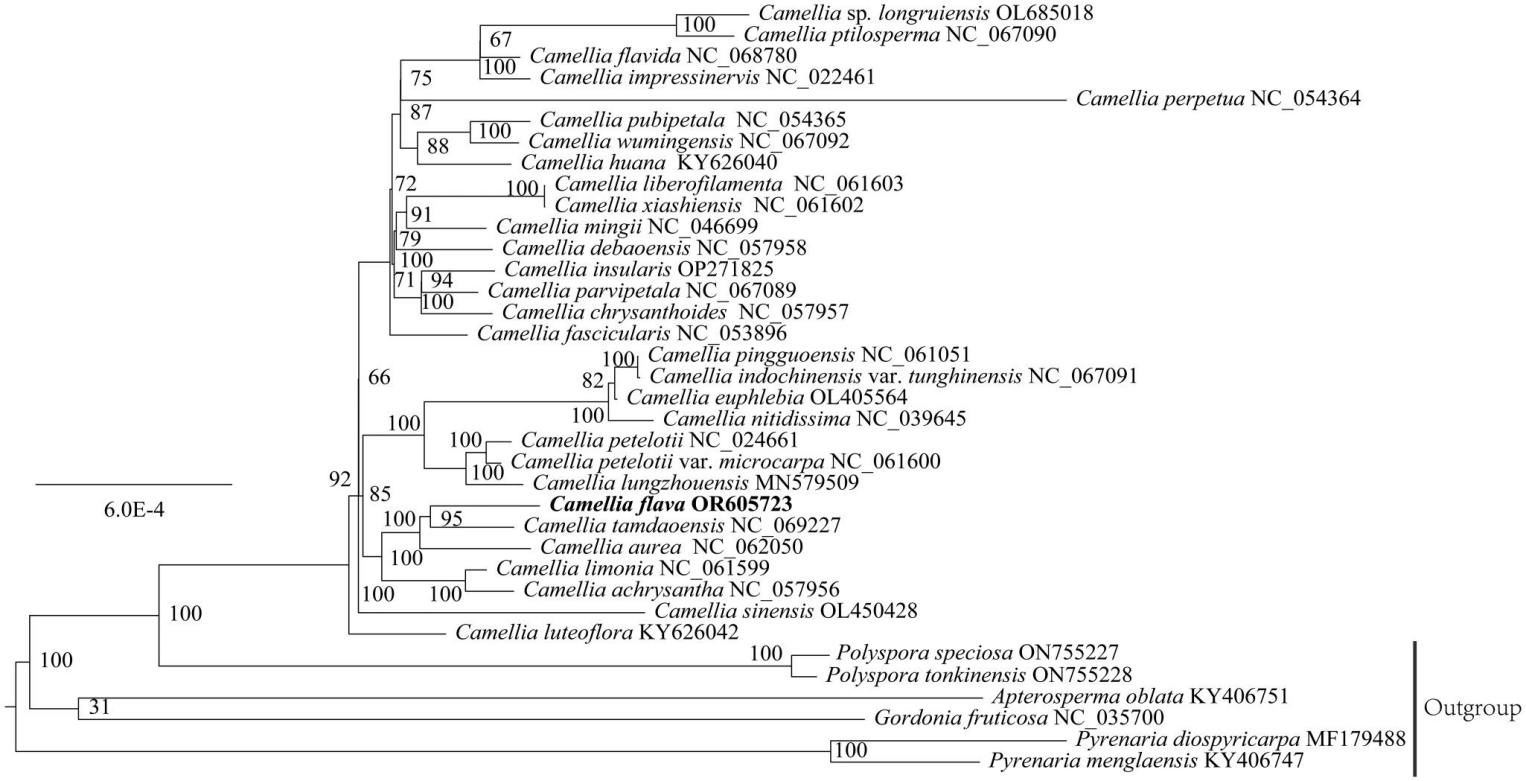
The Phylogenetic tree is constructed based on maximum likelihood, the scale represents the number of nucleotide substitutions at each site, and the number on each node represents 1000 ML bootstrap values, using the following sequence: *Camellia* sp. *longruiensis* OL685018, *C. sinensis* OL450428 (Chen et al. 2023), *C. ptilosperma* NC_067090, *C. impressinervis* NC_022461 (Tang et al. 2023), *C. flavida* NC_068780, *C. perpetua* NC_054364 (Pei et al. 2020), *C. wumingensis* NC_067092, *C. pubipetala* NC_054365 (Fan et al. 2021), *C. huana* KY626040 (Wang et al. 2017), *C. liberofilamenta* NC_061603 (Wang et al. 2017), *C. xiashiensis* NC_061602 (Ding et al. 2022), *C. mingii* NC_046699 (Zhang et al. 2019), *C. debaoensis* NC_057958 (Zheng and Wei 2021), *C. insularis* OP271825, *C. parvipetala* NC_067089, *C. chrysanthoides* NC_057957 (Lai and Tang 2021), *C. fascicularis* NC_053896 (Ding et al. 2022), *C. pingguoensis* NC_061051, *C. indochinensis* var. *tunghinensis* NC_067091, *C. euphlebia* OL405564, *C. nitidissima* NC_039645, *C. petelotii* NC_024661 (Huang et al. 2014), *C. petelotii* var. *microcarpa* NC_061600, *C. lungzhouensis* MN579509 (Fan et al. 2021), *C. tamdaoensis* NC_069227, *C. aurea* NC_062050, *C. limonia* NC_061599 (Ding et al. 2022), *C. achrysantha* NC_057956 (Lai and Tang 2021) and *C. luteoflora* KY626042 (Wang et al. 2017). *Polyspora speciosa* ON755227, *Po. tonkinensis* ON755228, *Apterosperma oblata* KY406751 (Yu et al. 2017), *Gordonia fruticosa* NC_035700 (Ci et al. 2019), *Pyrenaria menglaensis* KY406747 (Yu et al. 2017) and *Py. diospyricarpa* MF179488 (Zhu et al. 2018) are used as outgroups.

The complete chloroplast genome of *C. flava* was 156,670 bp in length, with an average sequencing depth of 841.91× (Figure S1). Like most other reported chloroplast genomes, it had a typical quadripartite structure consisting of a large single-copy (LSC, 86,250 bp), a small single-copy (SSC, 18,292 bp) and two inverted repeats (IRs, 26,064 bp) (Figure 2). The total GC content of the chloroplast genome was 37.32%, and the GC content of LSC, SSC, and IR regions was 35.35%, 30.56%, and 42.94%, respectively. The genome contained 133 genes, including 88 protein-coding genes, 37 transfer RNA genes, and eight ribosomal RNA genes. Among them, nine protein-coding genes (*atpF, petB, petD, ndhA, ndhB, rpl2, rpl16, rpoC1, rps16*) and six tRNA genes (*trnA-UGC, trnG-GAU, trnG-UCC, trnK-UUU, trnL-UAA, trnV-UAC*) each contained an intron. Two genes (*clpP* and *ycf3*) had two introns (Figure S2), respectively. The *rps12* gene was a trans-splicing gene with three unique exons (Figure S3). Phylogenetic analysis based on *Theaceae* species revealed that *Camellia* was a monophyletic group and *C. flava* sister to *C. tamdaoensis*, exhibited high resolution with strongly supported values (bootstrap ≥ 95 and posterior probability = 1) (Figure 3). The monophyly of sect. *Chrysantha* tribe was revealed with high support values.

## Discussion and conclusion

In this study, the chloroplast genome of *Camellia flava* was successfully sequenced and assembled. Like most angiosperms, the complete chloroplast genome of *C. flava* exhibits a typical quadripartite structure, including a large single-copy region (LSC), a small single-copy region (SSC), and two inverted repeat regions (IRs). Through research and analysis, it was found that the structure and gene content of the *C. flava* genome were similar to other published sect. *Chrysantha* genomes (Ding et al. 2022), which indicated that the *C. flava* chloroplast genome maintained relative conservation in the evolutionary process. This conservatism provides a solid basis for further comparative genome analysis and functional studies. In the phylogenetic analysis, the close relationship between *C. flava* and *Camellia tamdaoensis* was revealed. This finding was highly supported, suggesting that the two species may have a relatively close common ancestor and have maintained many common traits throughout evolution.

The genetic resources generated in this study are of great significance for the biological research, conservation, and breeding of *C. flava*. Genomic data can identify genes associated with important traits, assist breeders in selecting good individuals, and help track the genetic diversity and evolutionary history of populations, providing a scientific basis for conservation. The results of this study also provide valuable molecular resources for the phylogenetic and evolutionary study of *C. flava* and its relatives. In the future, with the accumulation of more data, more extensive genomic analysis can be carried out to further elucidate the evolutionary mechanism and functional characteristics.

## Supplementary Material

Supplyment_Table1.xlsx

## Data Availability

The genome sequence data that support the findings of this study are openly available in GenBank of NCBl at https://www.ncbi.nlm.nih.gov/ under accession No. OR605723. The associated BioProject, SRA, and Bio-Sample numbers are PRJNA1089488, SRR28385168 and SAMN40540878, respectively.
